# Role of microRNA Pathway in Mental Retardation

**DOI:** 10.1100/tsw.2007.208

**Published:** 2007-11-02

**Authors:** Abrar Qurashi, Shuang Chang, Peng Jin

**Affiliations:** Department of Human Genetics, Emory University School of Medicine, Atlanta, GA, USA

**Keywords:** microRNA, mental retardation, fragile X syndrome, fragile X mental retardation protein (FMRP), local protein translation, dendrites, synaptic activity

## Abstract

Deficits in cognitive functions lead to mental retardation (MR). Understanding the genetic basis of inherited MR has provided insights into the pathogenesis of MR. Fragile X syndrome is one of the most common forms of inherited MR, caused by the loss of functional Fragile X Mental Retardation Protein (FMRP).

MicroRNAs (miRNAs) are endogenous, single-stranded RNAs between 18 and 25 nucleotides in length, which have been implicated in diversified biological pathways. Recent studies have linked the miRNA pathway to fragile X syndrome. Here we review the role of the miRNA pathway in fragile X syndrome and discuss its implication in MR in general.

Mental retardation (MR) is defined as a failure to develop cognitive abilities and achieve an intelligence level that would be appropriate for the particular age group. About 2–3% of the total population is reported to be function two standard deviations below the mean IQ of the general population: below 70. Mild MR (IQ between 50 and 70) is most frequent (up to 80–85% of all MR) and is often associated with lower socioeconomic status, whereas the more severe forms occur in all social groups and in families of all educational levels. Understanding the molecular basis of MR would provide insights into human cognition and intelligence.

One of the most common forms of inherited MR is fragile X syndrome, which has an estimated prevalence of about 1 in 4,000 males and 1 in 8,000 females. The syndrome is transmitted as an X-linked dominant trait with reduced penetrance (80% in males and 30% in females). The clinical presentations of fragile X syndrome include mild to severe MR, with IQ between 20 and 70, mild abnormal facial features of a prominent jaw and large ears, mainly in males, and macro-orchidism in postpubescent males. In 1991, its molecular basis was revealed by positioning cloning and shown to be associated with a massive trinucleotide repeat expansion within the gene Fragile X Mental Retardation-1 (FMR1). Loss of the Fragile X Mental Retardation Protein (FMRP) has been identified as the major cause of fragile X syndrome. FMRP, as an RNA binding protein, has been implicated in translational control. Recently, FMRP has also been linked to the microRNA (miRNA) pathway that is involved in translational suppression. Here we will review the current knowledge on the biological functions of FMRP, and discuss the role of the miRNA pathway in the pathogenesis of fragile X syndrome and MR in general.

## MICRORNAS: BIOGENESIS AND MODE OF ACTION

miRNAs are endogenous, single-stranded RNAs between 18 and 25 nucleotides in length. The biogenesis of miRNAs involves enzymatic machinery that is well conserved from animals to plants. The transcription of miRNA genes is mainly directed by RNA polymerase II, which produces primary miRNAs (pri-miRNAs). In the nucleus, the RNase III endonuclease *Drosha*, along with DGCR8, excises pre- from pri-miRNA. The pre-miRNA is transported out of the nucleus by Exportin 5. In the cytoplasm, pre-miRNA is processed by *Dicer*, unwound, and loaded onto the effector complex, RISC (RNA-induced silencing complex), which then directs sequence-specific mRNA degradation or translational suppression (Fig. 1)[1,2].

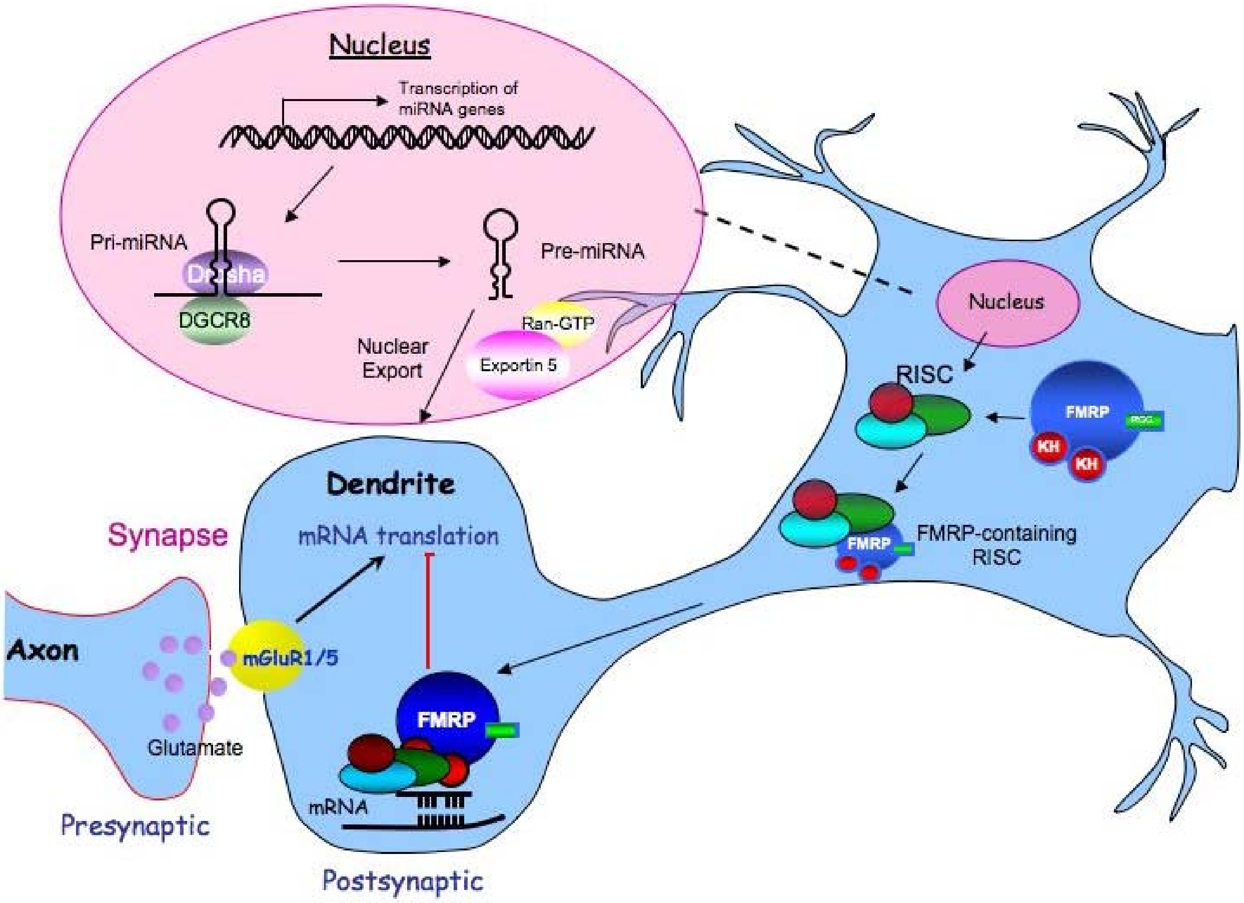

**FIGURE 1.** A model for FMRP-mediated translational suppression of mRNA targets via the miRNA pathway in the neuron. In the nucleus, miRNA genes are transcribed to generate primiRNAs, which are processed into the hairpin-structured pre-miRNAs by Drosha along with DGCR8. After being transported to the cytoplasm by Exportin 5, pre-miRNAs are further processed by Dicer to generate the duplex miRNA. Upon unwinding, one strand of the duplex is preferentially integrated into the RNA-induced silencing complex (RISC) and directs it to mRNA targets. FMRP forms a messenger ribonucleoprotein (mRNP) complex by interacting with specific RNA transcripts and proteins. At least some of the FMRP-mRNP complexes interact with RISC, which could regulate protein synthesis in the cell body of the neuron. In addition, FMRP-containing RISC could also be transported into dendrites to regulate local protein synthesis of specific RNAs in response to synaptic stimulation signals such as metabotropic glutamate receptor activation.

miRNAs function as negative regulators of gene expression by targeting mRNAs for translational repression, degradation, or both. In plants, miRNAs usually trigger mRNA degradation by perfect base pairing to its target mRNAs, while in animals, miRNAs usually inhibit translation through imperfect base pairing[3,4,5,6]. Recent data suggest that one miRNA could regulate multiple mRNA targets, while a given mRNA could be regulated by multiple miRNAs, which could provide transient and temporal specificities to translational regulation[6]. There has been a feverish increase in the identification and characterization of miRNAs and current data suggest that miRNAs regulate diversified biological pathways.

## MIRNAS IN NEURAL DEVELOPMENT

Recent years have seen an enormous increase in the list of neuronal-specific and -enriched species of miRNAs. Numerous studies showing the miRNA expression profile from different tissues and cellular systems have come up with a consensus that the brain displays 70% of experimentally detectable miRNAs, and among those with tissue-specific/enriched expression patterns, half are brain-specific/enriched[6]. During the course of development, miRNA expression profiles vary dynamically and temporally, as seen during the brain development in rodents[7,8,9], during the differentiation of primary neurons into mature differentiated neurons[10], or during differentiation of P19 embryonic carcinoma cells into neurons by retinoic acid treatment[11].

Despite the identification of so many miRNAs specifically expressed and enriched in the nervous system, unfortunately, direct functional studies of these miRNAs in mammalian nervous system are still sparse, and most conclusions are drawn from the studies made on lower model systems[12,13,14,15,16,17,18,19,20,21,22,23,24]. Studies in *C. elegans* have shown that cell fate decision between two taste receptor neurons and maintenance of their identity arises by a complex regulatory network of interactions between two miRNAs, miR-273 and lsy-6, and their transcription factor targets[25,26,27]. Using loss-of-function analysis, Gao and colleagues demonstrated that miR-9a is required to regulate precise production of neuronal precursor cells in the peripheral nervous system of *Drosophila* embryos and in adults[28]. Interestingly, miR-9a is conserved in humans and is highly expressed in fetal brains, thus suggesting that a similar mechanism may operate in mammalian neurogenesis as well. Furthermore, *Zebrafish* lacking maternal and zygotic components of Dicer, the enzyme necessary for the biogenesis of miRNAs, produces latter cellular differentiation defects notably in brain, which are specifically rescued by reintroduction of miR-430[29]. In a similar study, severe defects were observed in *Dicer*-deficient mice, which died very early in development and were depleted of pluripotent stem cells[30]. These two studies together suggest a crucial role of miRNAs in early embryonic patterning and morphogenesis.

There is a generalized view that *de novo* synthesis of proteins is required for neuronal construction and plasticity that ultimately affects higher brain functions, such as learning and memory. Various mRNAs have been shown to be transported actively and rapidly to dendrites in the form of transport-competent RNP particles. Once mRNAs arrive at their dendritic destination site, in the default state, translation is assumed to be repressed. However, in response to synaptic activity, appropriate spatiotemporal control of its translation is achieved, producing long-lasting changes in the synaptic strength that, in turn, are responsible for producing long-term memory[31]. Such local translational control is implemented at different levels and through various pathways. Interestingly, miRNA pathways have been recently identified in providing such a control. Kunes and colleagues[32] found that in dendrites of *Drosophila* neurons, mRNA translation for CaMKII-alpha (calmodulin-dependent protein kinase II) occurs in response to olfactory learning. The 3'-UTR of CaMKII-alpha contain two putative miRNA binding sites, indicating that binding of miRNAs might function to repress translation. Indeed, they showed the presence of miRNA processing machinery at synapses and further found that one of these proteins, encoding one of RISC components, Armitage, was degraded at the synapse during learning. When Armitage is degraded in response to neuronal activity, CaMKII-alpha mRNA translation increases. Taken together, these studies suggest that persistent generation of miRNAs is required to repress translation and that neuronal activity leads to degradation of components of the RNA interference machinery such that miRNAs are no longer generated, thereby lifting translational repression. In mammals, Greenberg and colleagues[21,33] investigated the expression and localization of candidate miRNAs in rat hippocampus, and revealed that miR-134 was specifically located close to synaptic sites in dendrites and enriched in synaptoneurosomal preparations. Furthermore, they found that miR-134 could regulate the translation of the mRNA encoding for LimKinase 1 protein (LimK1), which regulates actin filament dynamics and has an important role in dendrite and spine development and maintenance[31]. In another study, using rat primary cortical neurons, it was shown that miR-132 targets and represses P250GAP, a Rho/Rac regulator[24]. Interestingly, the activities of both miR-134 and miR-132 were shown to be modulated in response to synaptic activity. These studies suggest that the miRNA pathway is involved in the regulation of local protein synthesis.

## MIRNA PATHWAY IN MR: A CASE STUDY WITH FRAGILE X SYNDROME

### Neurobiology of Fragile X Syndrome

Fragile X syndrome is caused by the loss of functional FMRP. Since the molecular basis of fragile X syndrome was revealed by positioning cloning, a lot of effort has been dedicated to understanding the biological functions of FMRP and how the loss of FMRP leads to MR. FMRP belongs to a small family of highly conserved proteins referred to as the fragile X–related proteins[34,35]. Proteins of this family are characterized by the presence of two RNP K homology domains (KH domains), a cluster of arginine and glycine residues (RGG box), and both nuclear localization and nuclear export signals (Fig. 1)[36]. The KH domain and RGG box are common among RNA binding proteins. Indeed, FMRP has been found to bind RNA homopolymers and mRNAs *in vitro,* indicating a potential role for FMRP in the regulation of RNA metabolism[37]. Association of FMRP with polyribosomes in an RNA-dependant manner via mRNP particles has, in fact, been demonstrated. Both *in vitro* and *in vivo* studies have established a role for FMRP in translational regulation[37,38,39,40]. Using a variety of *in vitro* and *in vivo* assays, a number of putative RNA targets have been defined, some of which are affected in abundance and/or subcellular distribution in FMRP knockout cells[41,42,43,44,45].

Normally, expression of FMRP is widespread, although not necessarily ubiquitous, being most abundant in the brain and testis[46]. In neurons, FMRP has been found localized within and at the base of dendritic spines in association with polyribosomes[39]. Dendritic spines are the postsynaptic compartments of most excitatory synapses in mammalian brains. There is growing evidence that induction of synaptic plasticity correlates with changes in the number and/or shape of dendritic spines[47]**.** Dendritic spines in fragile X patients and *Fmr-1* knockout mice are denser apically, elongated, thin, and tortuous[48]. This association is RNA dependent as well as microtubule dependent, indicating a role for FMRP in mRNA trafficking and dendritic development (Fig. 1). Support for a role of FMRP in neural dysgenesis has been provided in studies identifying MAP1B, a key component of microtubule stability, as an mRNA target of FMRP. Lack of FMRP in *Fmr1* knockout mice was found to cause elevated levels of MAP1B protein and increased microtubule stability in neurons. Thus, loss of FMRP results in altered microtubule dynamics that affect neural development and, therefore, indicates a potential role for FMRP in synaptic plasticity[49,50]. Indeed, a link between abnormal dendritic spines and MR has been suggested for other MR diseases like Down syndrome and Rett syndrome[51,52]. The fact that spine defects are associated with MR in humans thus lends support to the view that local translation is a pivotal event in the cognitive processes.

A role for FMRP in synaptic plasticity, particularly long-term depression (LTD), seems logical since LTD is a protein synthesis–dependent phenomenon. Indeed, such a role for FMRP has been established. Activation of the metabotropic glutamate receptor 5 (mGluR5) by DHPG agonist stimulates LTD under normal conditions. LTD activated in this manner requires new protein synthesis, but does not require transcription[53]. These data suggest that mGluR5 stimulation allows for the removal of translational repression of transcribed and localized mRNA messages necessary for LTD. Fitting with such a model is the observation that mGluR-dependent LTD is exaggerated in an *Fmr1* knockout mouse[54]. It has been suggested that the presence of FMRP represses translation of proteins involved in LTD and that stimulation of mGluR5 results in the localized translation of these transcripts. Currently, the effect of mGluR5 antagonists on the balance between FMRP-mediated translational repression and mGluR5-activated protein synthesis is seen as potential target for new therapies treating fragile X syndrome.

## miRNA Pathway in Fragile X Syndrome

Initial biochemical studies in *Drosophila* to identify protein components of *dFmrp*-containing complexes and RISC led to the identification of *dFmrp* as a component of RISC. Further analysis revealed specific interactions between *dFmrp* and two functional RISC proteins, *dAGO2* and *Dicer*[55,56]. Although *dAGO2* is generally associated with siRNA-mediated gene silencing in *Drosophila*, the loss of *dFmr1* does not seem to affect RNAi[55,56,57]. In addition, the endogenous miRNAs have been found associated with FMRP in both flies and mammals[58]. Therefore, it has been proposed that FMRP-mediated translational suppression occurs via the miRNA pathway and involves miRNA. This view is further supported by the association of FMRP with a mammalian Argonaute, eIF2C2, which is itself a component of miRNA-containing mRNP complexes. In adult mice brains, Dicer and eIF2C2 have also been observed to interact with Fmrp at postsynaptic densities[58,59,60].

Importantly, the genetic interactions between *dFmr1* and the components of the miRNA pathway have been demonstrated in *Drosophila*[59]. *dAGO1* was shown to interact dominantly with *dFmr1* in *dFmr1* loss-of-function and overexpression models[59]. Overexpression of *dFmr1* leads to a mildly rough eye phenotype that is the result of increased neuronal cell death. By introducing a recessive lethal allele of *AGO1*, containing a P-element insertion that reduces its expression level, suppression of the mild rough eye phenotype could be observed. The loss-of-function model revealed that *AGO1* was required for *dFmr1* regulation of synaptic plasticity. In the absence of *dFmr1*, pronounced synaptic overgrowth can be observed in the neuromuscular junction (NMJ) of *Drosophila* larvae, similar to the dendritic overgrowth seen on the brains of *Fmr1* knockout mice and human patients. It was shown that while both *dFmr1* and *dAGO1* heterozygotes had normal NMJs, a transheterozygote displayed strong synaptic overgrowth and overelaboration of synaptic terminals[58,59]. This result suggests that a limiting factor for function of *dFmr1* at synapses is the function of *AGO1*. Rather significantly, this directly implicates the potential role of the miRNA pathway in human disease since *dAGO1* modulates translational suppression mediated by *dFmrp*. The genetic interaction between *dFmr1* and *dAGO2* has also been demonstrated in experiments concerning the gene *pickpocket1* (*ppk1*), which was found to control rhythmic locomotion in flies. *ppk1* was found to be an mRNA target of *dFmrp*, and the expression of PPK1 seems to be regulated by both *dFmr1* and *dAGO2*[61].

Another recent study showing the involvement of *dFmrp* in miRNA/RNAi function comes from the identification of P body–like granule in *Drosophila* neurons[63]. A set of neuronal Staufen-containing RNPs that function in the transport and translational control of mRNAs share fundamental organization and function with maternal RNA granules and somatic P bodies. Many proteins found in P bodies, including the RNA-degradation enzymes (Dcp1p and Xrn1p/Pacman), components of miRNA (Argonaute), nonsense-mediated decay (NMD [Upf1p]), and general translational repression (Dhh1p/Me31B) pathway have been identified in them. Interestingly, Me31B/Dhh1p present in P body–like granule containing Staufen and dFMR1 functions together with another dFMR1-associated, P body protein (trailerhitch/Scd6p) in dFMR1-driven, Argonaute-dependent translational repression in dendritic morphogenesis and miRNA function *in* vivo[63].

Experiments concerning the role of FMRP-KH2 in binding “kissing complex” RNA for FMRP-mediated translational suppression may hint at a mechanism by which FMRP cooperates with the miRNA pathway. The *in vitro* RNA selection experiments were able to identify “kissing complex” as a structure that specifically interacts with the KH2 domain of FMRP and prevents association of FMRP with polyribosomes[62]. However, it is unclear whether the KH2 domain of FMRP binds to inter- or intramolecular “kissing complexes”. Since the *in vitro* RNA selection experiments were performed with random RNA libraries, it remains possible that the “kissing complex” identified represents an intermolecular interaction, whereby FMRP-KH2 is stabilizing a duplex between a miRNA and an mRNA target, which would, therefore, suppress translation of that target[62]. Although the current data strongly support the idea that FMRP could regulate the translation of its mRNA through miRNA interaction, unfortunately, the exact mechanism of its action together with RISC is not clear at present. However, one could envisage some reasons for this kind of mechanism. Association of miRNA, FMRP, and RISC could combine and modify their classical properties to emerge as a single entity with dynamic, quick, and reversible features suitable for local protein synthesis–dependent synaptic plasticity. For example, FMRP has an intrinsic property to discriminate among different RNAs, which leads to that RISC interacts with different combinations of RNA. RISC core, in turn, could modify or diversify the translational ability of FMRP.

While, AGO proteins and FMRP have been generally thought to mediate translational repression, studies from Vasudevan and Steitz[64] have given another complexity in the mechanism of translation. Under specific cellular conditions, Argonaute 2 (AGO2) and fragile X mental retardation–related protein 1 (FXR1), can also act together as translation activators. Both AGO2 and FXR1 simultaneously bind to the AU-rich element in the 3'-UTR of TNF-alpha mRNA, and activates its translation in a cell growth-dependent manner[64]. In this context, it will be interesting to determine whether previously identified complexes of AGO proteins and FMRP form similar complexes to up-regulate translation of mRNAs, and if so, what are their partners?

## CONCLUDING REMARKS

The accumulated data from genetic, biochemical, and molecular studies suggest that FMRP could utilize the miRNA pathway to regulate the translation of specific mRNAs, particularly in local protein synthesis at dendrites. Given the link between abnormal dendritic spines and MRs, it is very likely that the misregulation of the miRNA pathway could contribute to the disease pathogenesis of MR in general, and it would be interesting and important to examine further other types of inherited MR, such as Rett syndrome and Down syndrome, and determine the potential involvement of the miRNA pathway in disease pathogenesis.
